# Cerebrospinal Fluid Pulsation Stress Promotes the Angiogenesis of Tissue-Engineered Laminae

**DOI:** 10.1155/2020/8026362

**Published:** 2020-07-02

**Authors:** Linli Li, Yiqun He, Han Tang, Wei Mao, Haofei Ni, Feizhou Lyu, Youhai Dong

**Affiliations:** ^1^Department of Orthopedics, Shanghai Fifth People's Hospital, Fudan University, China; ^2^Department of Orthopedics, Huashan Hospital, Fudan University, China

## Abstract

**Background:**

Angiogenesis is a prerequisite step to achieve the success of bone regeneration by tissue engineering technology. Previous studies have shown the role of cerebrospinal fluid pulsation (CSFP) stress in the reconstruction of tissue-engineered laminae. In this study, we investigated the role of CSFP stress in the angiogenesis of tissue-engineered laminae.

**Methods:**

For the *in vitro* study, a CSFP bioreactor was used to investigate the impact of CSFP stress on the osteogenic mesenchymal stem cells (MSCs). For the *in vivo* study, forty-eight New Zealand rabbits were randomly divided into the CSFP group and the Non-CSFP group. Tissue-engineered laminae (TEL) was made by hydroxyapatite-collagen I scaffold and osteogenic MSCs and then implanted into the lamina defect in the two groups. The angiogenic and osteogenic abilities of newborn laminae were examined with histological staining, qRT-PCR, and radiological analysis.

**Results:**

The *in vitro* study showed that CSFP stress could promote the vascular endothelial growth factor A (VEGF-A) expression levels of osteogenic MSCs. In the animal study, the expression levels of angiogenic markers in the CSFP group were higher than those in the Non-CSFP group; moreover, in the CSFP group, their expression levels on the dura mater surface, which are closer to the CSFP stress stimulation, were also higher than those on the paraspinal muscle surface. The expression levels of osteogenic markers in the CSFP group were also higher than those in the Non-CSFP group.

**Conclusion:**

CSFP stress could promote the angiogenic ability of osteogenic MSCs and thus promote the angiogenesis of tissue-engineered laminae. The pretreatment of osteogenic MSC with a CSFP bioreactor may have important implications for vertebral lamina reconstruction with a tissue engineering technique.

## 1. Background

Tissue engineering techniques have been successfully used to repair the vertebral lamina defect. The reconstructed artificial laminae can reconstruct the posterior column structure of the spine, effectively reducing the occurrence of epidural scar tissue, nerve root adhesion, and spinal degradation [1, 2].

Previous studies have investigated the effect of biological and mechanical factors on the formation of the artificial vertebral laminae. The result revealed that the biological factors released from the bone end could initiate the early onset osteogenesis of the artificial laminae, and the mechanical stimulation of cerebrospinal fluid pulsation (CSFP) stress could promote the osteogenesis and remodeling of the artificial laminae [3, 4].

When bone defects are repaired using tissue engineering technology, angiogenesis is a prerequisite step to achieve the success of bone regeneration [5]. Many strategies have been used to enhance vascularization of tissue-engineered bone, such as a specific scaffold design, the addition of stem cells or angiogenic factors, *in vitro* prevascularization of tissue-engineered bone, and *in vivo* prevascularization [6–13]. Mesenchymal stem cells (MSCs) are among the most promising stem cell types for vascular tissue engineering and have been widely used among all the above-mentioned strategies [6, 7, 9], because MSCs not only can transdifferentiate into all cell lineages of three germ layers including blood vessel cells arising from mesodermal tissue but also can secrete a series of angiogenic factors, such as vascular endothelial growth factor (VEGF), Monocyte Chemoattractant Protein 1 (MCP-1), Interleukin (IL-6), exosomes, and miRNAs [6, 7]. The expression levels of VEGF-A also elevated during MSC osteogenesis [14]. Previous studies have shown that mechanical stimulations, especially the fluid shear stress and cyclic strain, could induce MSCs towards vascular differentiation and MSCs to express angiogenic factors [7]. But few studies are investigating the role of pulsation stress, especially the CSFP stress, on the angiogenic abilities of MSCs.

**Figure 1:**
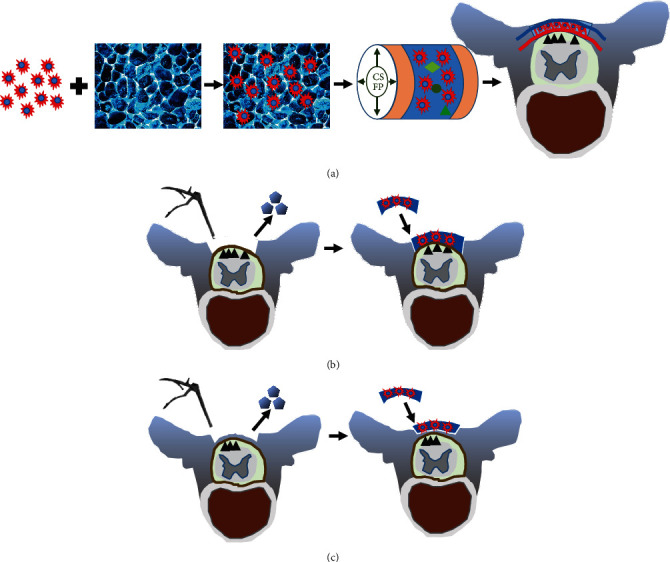
(a) Schematic illustration of the effects of CSFP stress on the angiogenesis of tissue-engineered laminae. (b) The diagrammatic sketch of the CSFP animal model. The native laminae were removed by rongeur, and then, the TEL was implanted into the laminae defect. (c) The diagrammatic sketch of the Non-CSFP animal model. The outer cortex of the native laminae was removed by rongeur, and then, the tissue-engineered laminae (TEL) was implanted onto the inner cortex.

CSFP is a continuous pulsation stress caused by heartbeat and respiration, and it changes with cardiac and respiratory rhythms [15]. In rabbits, the spinal dura mater pumped like a blood vessel with cerebrospinal fluid flowing inside, creating the cerebrospinal fluid pulsation stress. And the previous study confirmed that the CSFP stress at the lumbar vertebrate ranged from 10 to 20 mm water pressure, with a frequency of 3-4 Hz [3]. Therefore, we made a speculation that the CSFP stress could promote the angiogenic factor expressions of MSCs and then promote the angiogenesis of tissue-engineered laminae (Figure 1(a)).

Thus, in this study, we aimed to investigate the role of CSFP stress on the angiogenic ability of tissue-engineered laminae (TEL) constructed by osteogenic MSCs and hydroxyapatite-collagen I scaffold. For the *in vitro* study, we used the CSFP bioreactor to investigate the impact of CSFP stress on the osteogenic Wharton jelly mesenchymal stem cells (MSCs). For the *in vivo* study, we implanted TEL into the lamina defect site in both CSFP and Non-CSFP groups. Then, the angiogenic and osteogenic abilities of newborn laminae were examined with histological staining, qRT-PCR, and radiological analysis for up to 12 weeks postimplantation.

## 2. Materials and Methods

### 2.1. Intervention of Osteogenic MSCs in the CSFP Bioreactor

The MSCs we used in this study were isolated from rabbit umbilical cord Wharton's jelly. The isolation, culture, and osteogenic differentiation of MSCs were described in the previous article [3]. After 14 days of culture in the osteogenic medium (containing 10 nM dexamethasone, 10 mM *β*-glycerophosphate sodium, 50 mg/ml ascorbic acid, and 10 nM 1,25-dihydroxy vitamin D3, Sigma-Aldrich, USA), the osteogenic WJ-MSCs were used for the following experiments.

Under the sterile condition, the PLGA scaffolds (Nuoqi, Chongqing, China) were cut to the size of 40 mm × 15 mm × 0.1 mm. The osteogenic MSCs were trypsinized and resuspended in media at a concentration of 1 × 10^6^/ml. Then, 100 *μ*l cell suspension was pipetted on one side of each scaffold and cultured in Petri dishes in the incubator at 37°C under a 5% CO_2_ atmosphere. After 2 hours, the osteogenic medium was added, and all constructs were placed in the osteogenic medium for 8 hours before being fixed in the bioreactor.

**Figure 2:**
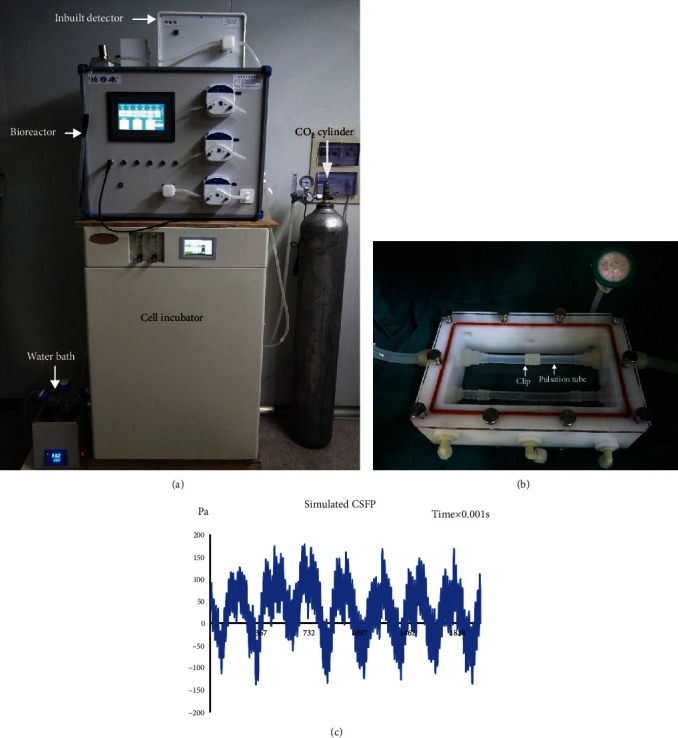
Cerebrospinal fluid pulsation bioreactor system. (a) The modules of the bioreactor system. (b) The response chamber, which was placed in the cell incubator, and the cell sheets were placed between the clip and the pulsation tube. (c) The simulated waves of CSFP stress by the bioreactor system.

The setting condition of the CSFP bioreactor was as below: flow velocity, 6 cm/s; frequency, 3 Hz. For the CSFP+osteogenic MSC group, the constructs were fixed by the clip on the pulsation tube in the CSFP bioreactor. For the osteogenic MSC group, the constructs were fixed by the clip on the control tube in the CSFP bioreactor. And we used undifferentiated MSCs seeded on the PLGA scaffolds as the control group for qRT-PCR analysis. After 24 hours, the constructs were taken out for qRT-PCR analysis and immunofluorescence assay of VEGF-A (Figure 2).

### 2.2. Tissue-Engineered Lamina Construction

The protocol was approved by the Committee on the Ethics of Animal Experiments of Fudan University (No. 20150482A168). The hydroxyapatite-collagen I scaffold was bought from the Beijing Allgens Medical Science & Technology Co., Ltd. Under the sterile condition, the hydroxyapatite-collagen I scaffold was cut to the size of 10 mm × 8 mm × 1 mm. After 2 weeks of culture in osteogenic medium, osteo-differentiated MSCs were trypsinized and resuspended in media at a concentration of 1 × 10^6^/ml. Then, 100 *μ*l cell suspension was pipetted on one side of each scaffold, and after 30 min, 100 *μ*l cell suspension was pipetted on the other side. All constructs were placed in the osteogenic medium for another week before implantation.

In order to observe the cell activity on the scaffold, the osteogenic MSCs in the scaffolds were stained with live/dead (Thermo Fisher, USA) and the nucleus was counterstained with DAPI (Southern Biotech, USA). And the constructs were observed under a confocal microscope (ZEISS).

### 2.3. Construction of CSFP and Non-CSFP Rabbit Models

Forty-eight 2-month-old male rabbits weighing 2.25 ± 0.25 kg were randomly divided into the CSFP group (*n* = 24) and the Non-CSFP group (*n* = 24). The animals were anesthetized with pentobarbital sodium (1 ml/kg intraperitoneally). The surgical procedures of making rabbit models were described in below.

For rabbits in the CSFP group, we located the spinous process of fifth lumbar vertebrate by the anatomical landmark and made a 3 cm longitudinal skin incision. The superficial fascia and paraspinal muscle were retracted to expose the spinous process, which was then removed to expose the native laminae. At last, a bone defect measuring 10 mm × 8 mm was created on the native laminae by a rongeur to expose the dura. The removal of native laminae left two flesh cancellous bone ends measuring 10 mm × 2 mm. The TEL was fixed in the bone defect (Figure 1(b)).

For rabbits in the Non-CSFP group, we removed the paraspinal muscle with a detacher to expose the spinous process and laminae and then removed the spinous process by a rongeur. By removing the spinous process, a cancellous bone end measuring 10 mm × 4 mm was created, similar to that of the CSFP group (10 mm × 2 mm × 2 ), while preserving the dura surface cortex of laminae. The TEL was fixed onto the lamina defect site (Figure 1(c)).

### 2.4. Immunofluorescence Assay

For the MSC-PLGA constructs, the constructs were fixed with 3% paraformaldehyde in PBS for 10 min. Nonspecific binding was then reduced by incubating cells with 5% BSA (Gibco, USA) for 30 min. VEGF-A was then labeled with its specific primary antibody (Abcam; cat. no. ab1316; 5 *μ*g/ml) for 12 hours at 4°C. Cells were then washed with PBS and incubated with rabbit anti-mouse horseradish peroxidase-conjugated secondary antibodies (Jackson ImmunoResearch Laboratories, Inc. USA; dilution, 1 : 100) for 1 hour and DAPI Fluoromount G (Southern Biotech, USA) for 5 min. Each sample was then imaged using a fluorescence inversion microscope system (Leica, Germany) with dual excitations.

### 2.5. Micro-CT (Computed Tomography) Examination

The tissue specimens were harvested in the 2^nd^, 4^th^, 8^th^, and 12^th^ weeks after implantation and were immediately fixed in freshly prepared 4% (*w*/*v*) paraformaldehyde. The osteogenesis of specimens was examined using micro-CT at the Shanghai Public Health Clinical Center. The 3D model was reconstructed manually using the GEHC MieroView2.0+ABA software. The threshold was set at 1000 for all the samples, except the sample of the Non-CSFP group at the 2^nd^ week (threshold = 500).

### 2.6. Histological Staining

After the micro-CT examination, the tissue specimens were decalcified with 10% ethylenediaminetetraacetic acid for 4 weeks. Tissue sections with 6 *μ*m thickness were cut on a microtome and mounted onto glass slides. The sections were processed for routine histological analysis by hematoxylin-eosin (HE) staining and immunohistochemistry (IHC) staining.

### 2.7. IHC Staining

After baking for 2 h at 60°C, the sections were dewaxed with xylenes and then rehydrated through a series of graded ethanol to distilled water. Endogenous peroxidase activity was blocked with 0.3% hydrogen peroxide for 30 min at 37°C. For antigen retrieval, the sections were submerged in sodium citrate buffer (pH 6.0) for 10 min and then incubated with normal goat serum for 30 min at 37°C to reduce the nonspecific binding. The mouse anti-PECAM1 (Invitrogen; cat. no. MA5-13188), anti-bFGF (Abbiotec, cat. no. 250559), and anti-BMP2 (Abcam, cat. no. ab6285) were applied at the dilution of 1 : 50 overnight at 4°C and followed by incubation with horseradish peroxidase- (HRP-) conjugated secondary antibodies (Changdao, China). After rinsing, staining was performed with DAB and counterstained with hematoxylin to display the nucleus. Each slide was imaged using the inversion microscope system (Leica, Germany). The images were analyzed by the software of Image J.

For immunofluorescence assay, the processing of the slides was the same as the above. PECAM1 was then labeled with its specific primary antibody (Servicebio; cat. no. GB13063; dilution, 1 : 50) for 12 hours at 4°C. Then, the sections were washed with PBS and incubated with donkey anti-rabbit horseradish peroxidase-conjugated secondary antibodies (Servicebio; cat. no. GB21404; dilution, 1 : 300) for 1 hour and DAPI (Servicebio; cat. no. G1012) for 10 min. The slides were sealed with antifluorescence quenching sealant. Each slide was imaged using a fluorescence inversion microscope system (Leica, Germany) with dual excitations.

### 2.8. Quantitative Real-Time PCR

For MSC-PLGA constructs, the total RNA was extracted from the constructs using TRIzol® Reagent (Life Technologies, USA). All RNA samples were then treated with RNase-free DNase I (Qiagen, Valencia, CA) to digest the genomic DNA. Aliquots of 500 ng total RNA were reverse transcribed to cDNA using the PrimeScriptTM RT Master Mix (TaKaRa, Japan). Quantitative real-time PCR (qRT-PCR) was performed using a 7900 Real-Time PCR System (Applied Biosystems) with the Power SYBR Green PCR Master Mix (Applied Biosystems, Warrington, UK). The relative gene expression was calculated using the following equation: ΔCt = Ct (VEGF-A) -Ct (*ACTB*); ΔΔCt = ΔCt (CSFP+osteogenic MSCs/osteogenic MSCs) -*Δ*Ct (undifferentiated MSCs); fold change = 2^−ΔΔCt^.

For the animal study, tissue specimens were harvested in the 2^nd^, 4^th^, 8^th^, and 12^th^ weeks after implantation and immediately immersed in the TRIzol® Reagent (Life Technologies, USA). Native laminae were also harvested in the 0^th^ week as the normal laminae. The tissue specimens were then grounded until there was no obvious tissue residual. Total RNA was extracted from the grounded tissue using TRIzol® Reagent. The following procedures were the same as mentioned above. The relative gene expression was calculated using the following equation: ΔCt = Ct (test genes) -Ct (*ACTB*); ΔΔCt = ΔCt (artificial laminae) -*Δ*Ct (normal laminae); fold change = 2^−ΔΔCt^.

The gene-specific primers used for *PECAM-1*, *VEGF-A*, *OCG-3*, *Osterix*, and *ACTB* are listed in Table 1.

### 2.9. Statistical Analysis

Each experiment was repeated three times. Statistical analyses were performed using SPSS version 19.0 for Windows. One-way analysis of variance (ANOVA) was used to confirm comparisons of the variables. Significance was identified as a *p* value of less than 0.05. ∗ represents *p* values < 0.05.

## 3. Results

### 3.1. CSFP Bioreactor

**Figure 3:**
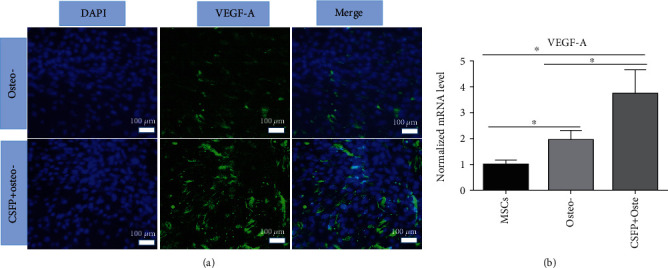
The VEGF-A expression of osteogenic WJ-MSCs with/without CSFP stimulation in the CSFP bioreactor system. (a) IF staining showed the CSFP+osteogenic WJ-MSC group had higher VEGF-A expression levels. (b) QRT-PCR assay showed the CSFP+osteogenic WJ-MSC group had higher VEGF-A mRNA expression levels.

The *VEGF-A* mRNA expression level in the CSFP+osteogenic MSC group was significantly higher than that in the osteogenic MSC group and the undifferentiated group. The IF staining of VEGF-A also showed that the CSFP+osteogenic MSC group had higher VEGF-A expression (Figure 3).

### 3.2. TEL Construction

**Figure 4:**
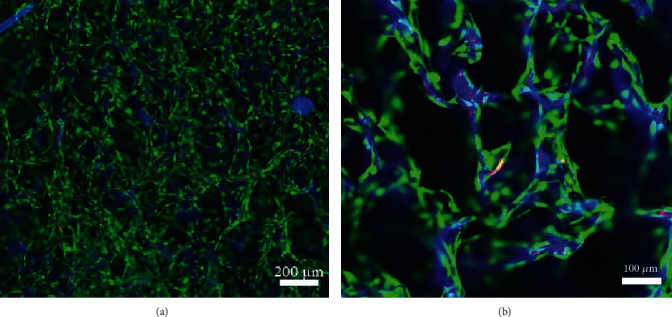
The live/dead staining of the TEL made by osteogenic and hydroxyapatite-collagen I scaffold. The scaffold had blue autofluorescence.

The live/dead staining showed that almost all the osteogenic MSCs survived 7 days after implantation into hydroxyapatite-collagen I scaffold, and the cells tightly adhered to the trabeculae structure of the scaffold. The scaffold has blue autofluorescence (Figure 4).

### 3.3. Micro-CT Examination

**Figure 5:**
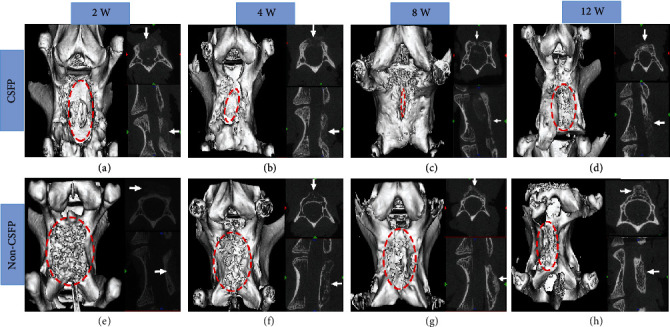
The 3D reconstruction pictures of the newborn laminae in the CSFP and Non-CSFP groups at the 2^nd^, 4^th^, 8^th^, and 12^th^ weeks. The dashed red oval in images (a), (b), and (c) showed the laminae defect, and the dashed red oval in other images showed the observation area.

In the CSFP group, the artificial laminae grew gradually from the two sides of bone ends to the middle, and the bone defect narrowed correspondingly. In the 12^th^ week, the artificial laminae on dural surface realized symphysis, with a similar arch and smoothness with the native laminae, while the artificial laminae on the paraspinal muscle surface had not realized symphysis (Figures 5(a)–5(d)).

In the Non-CSFP group, the ectopic artificial laminae grew from the paraspinal muscle side towards the native laminae side. From the 2^nd^ week to the 12^th^ week, the number of bone trabeculae on the paraspinal muscle surface of the artificial ectopic laminae increased gradually, while there was little trabecula formation on the native laminae side (Figures 5(e)–5(h) ).

### 3.4. HE Staining

**Figure 6:**
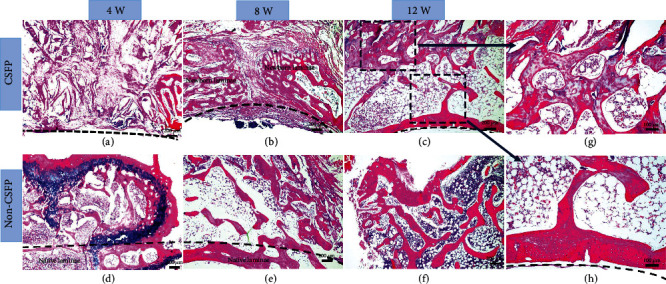
(a–f) Representative images of HE staining of newborn laminae in the CSFP group and the Non-CSFP group at the 4^th^, 8^th^, and 12^th^ weeks. (g) The paraspinal surface of the newborn laminae in the 12^th^ week. (h) The dural surface of the newborn laminae in the 12^th^ week. The dashed line in images (a), (b), (c), and (h) showed the vertebral canal. The dashed line in images (d) and (e) showed the outer cortex of native laminae.

In the CSFP group, the artificial laminae grew from the two sides of the bone ends to the middle. The bone growth rate on the dural surface was higher than that on the paraspinal muscle surface. In the 8^th^ week, the lamina defect was less than 200 *μ*m; the trabecula amount of the artificial laminae on the dural surface was higher than that on the paraspinal muscle surface (Figure 6(b)). In the 12^th^ week, the laminae on the dural surface were completely developed, showing similar trabecula structure and curvature to the native laminae; however, the artificial laminae on the paraspinal muscle surface were still undergoing osteogenesis (Figures 6(c), 6(g), and 6(h)).

In the Non-CSFP group, the artificial ectopic laminae grew from the paraspinal muscle side towards the native laminae side. The trabecula density and amount of the artificial ectopic laminae on the paraspinal muscle surface were higher than those on the native laminae side. From 4 weeks to 12 weeks, the number of trabeculae increased, but the arrangement of the trabecular bone was always disorganized (Figures 6(d)–6(f)). Besides, the osteogenesis process resembled endochondral ossification (Figure 6(d)).

### 3.5. Angiogenesis

The angiogenesis of the two groups was analyzed through the protein expression levels of PECAM-1 and bFGF and the mRNA expression levels of *PECAM-1* and *VFGF-A*. They showed similar expression trends.

**Figure 7:**
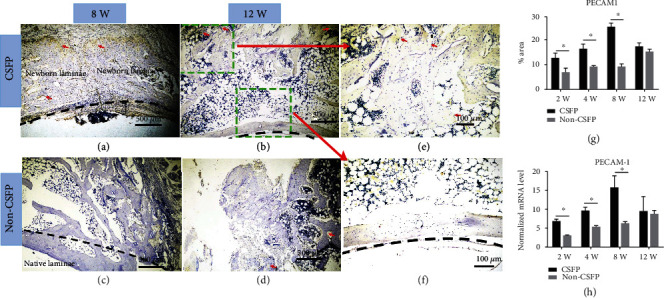
(a–d) Representative images of PECAM1 IHC staining of newborn laminae in the CSFP group and the Non-CSFP group at the 4^th^ and 8^th^ weeks. (e) The PECAM1 IHC staining of the paraspinal surface of the newborn laminae in the 12^th^ week. (f) The PECAM1 IHC staining of the dural surface of the newborn laminae in the 12^th^ week. (g) Quantification of PECAM1 expression by IHC staining. (h) QRT-PCR analysis of PECAM1 expressions. The red arrow showed positive staining. The dashed line in images (a), (b), and (f) showed the vertebral canal. The dashed line in images (c) showed the outer cortex of native laminae.

IHC staining showed that the expression levels of PECAM-1 in the CSFP group increased from the 2^nd^ week to the 8^th^ week and then decreased from the 12^th^ week, while the expression levels of PECAM1 in the Non-CSFP group increased slowly from the 2^nd^ week to the 12^th^ week, and the protein expression levels of PECAM1 in the CSFP group were significantly higher than those in the Non-CSFP group in the 2^nd^, 4^th^, and 8^th^ weeks (*p* < 0.05) (Figures 7(a)–7(g)).

**Figure 8:**
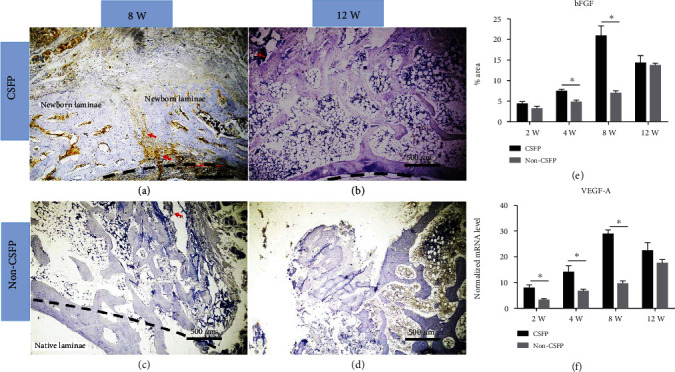
(a–d) Representative images of bFGF IHC staining of newborn laminae in the CSFP group and the Non-CSFP group in the 8^th^ and 12^th^ weeks. (e) Quantification of bFGF expression by IHC staining. (f) QRT-PCR analysis of *VEGF-A* expressions. The red arrow showed positive staining. The dashed line in images (a) and (b) showed the vertebral canal. The dashed line in images (c) showed the outer cortex of native laminae.

The protein expression levels of bFGF in the CSFP group increased from the 2^nd^ week to the 8^th^ week and then decreased from the 12^th^ week, while the expression levels of bFGF in the Non-CSFP group increased from the 2^nd^ week to the 12^th^ week; the expression levels of bFGF in the CSFP group were significantly higher than those in the Non-CSFP group in the 2^nd^ and 4^th^ weeks (*p* < 0.05). (Figures 8(a)–8(e)).

The mRNA expression trends of *PECAM-1* and *VFGF-A* were consistent with the protein expression trends of PECAM-1 and bFGF, and the mRNA expression levels of *PECAM-1* and *VFGF-A* in the CSFP group were significantly higher than those in the Non-CSFP group in the 2^nd^, 4^th^, and 8^th^ weeks (*p* < 0.05) (Figures 7(h) and 8(f)).

Moreover, in the CSFP group, the protein expression levels of bFGF on the dural surface were higher than those on the paraspinal muscle surface in the 8^th^ week (Figure 8(a)). In the 12^th^ week, the undeveloped artificial laminae on the paraspinal muscle surface showed higher expression levels of PECAM-1 than the developed artificial laminae on the dural surface (Figures 7(b), 7(e), and 7(f)).

**Figure 9:**
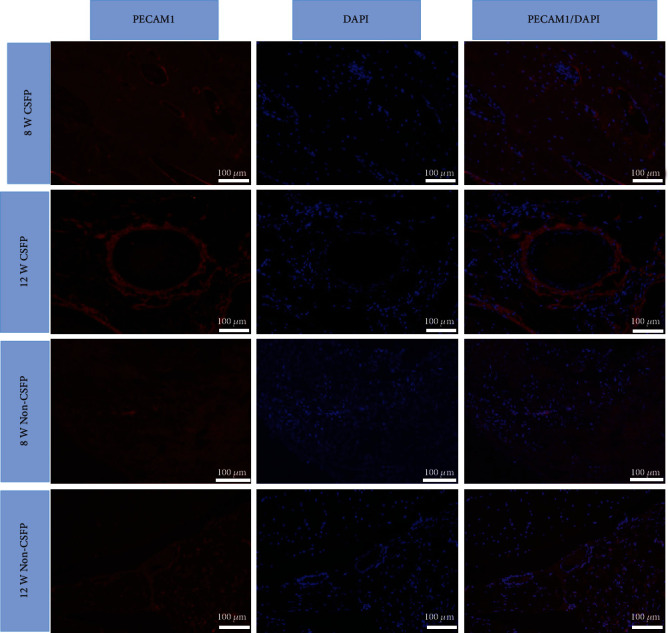
Immunofluorescence staining of PECAM1 of the newborn laminae in the CSFP group in the 8^th^ and 12^th^ weeks.

Immunofluorescence staining showed, in the CSFP group, an abundance of organized small vessels in the artificial laminae in the 8^th^ week, and the vessels became more developed in the 12^th^ week. In the Non-CSFP group, few vessels formed near the native laminae side in the ectopic artificial laminae in the 8^th^ week, and many small vessels formed near the paraspinal surface side in the ectopic artificial laminae at the 12^th^ week (Figure 9).

### 3.6. Osteogenesis

The osteogenesis of the two groups was analyzed through the protein or mRNA expression levels of the osteogenic markers of BMP-2, Osterix, and OCG-3.

**Figure 10:**
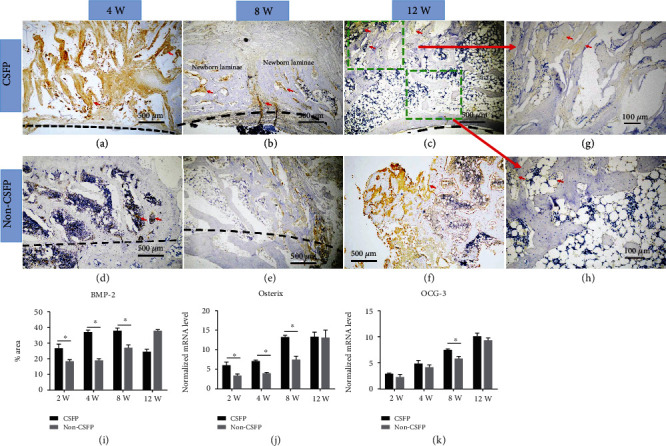
(a–c) Representative images of BMP-2 IHC staining of newborn laminae in the CSFP group and the Non-CSFP group in the 4^th^, 8^th^, and 12^th^ weeks. (g) The BMP-2 IHC staining of the paraspinal surface of the newborn laminae in the 12^th^ week. (h) The BMP-2 IHC staining of the dural surface of the newborn laminae in the 12^th^ week. (i) Quantification of BMP-2 expression by IHC staining. QRT-PCR analysis of *Osterix* (j) and *ODG-3* (k) expressions. The red arrow showed positive staining. The dashed line in images (a), (b), and (c) showed the vertebral canal. The dashed line in images (d) and (e) showed the outer cortex of native laminae.

IHC staining showed that the expression levels of BMP-2 in the CSFP group increased from the 2^nd^ week to the 8^th^ week and decreased in the 12^th^ week, while its expression levels in the Non-CSFP group increased slowly from the 2^nd^ week to the 12^th^ week, and the expression levels of BMP-2 in the CSFP group were significantly higher than those in the Non-CSFP group in the 2^nd^, 4^th^, and 8^th^ weeks (*p* < 0.05) (Figures 10(a)–10(f) and 10(i)).

The mRNA expression of the *Osterix* increased from the 2^nd^ week to the 12^th^ week, and the mRNA expression levels of the *Osterix* in the CSFP group were significantly higher than those in the Non-CSFP group in the 2^nd^, 4^th^, and 8^th^ weeks (*p* < 0.05) (Figure 10(j)). The mRNA expression levels of *OCG-3* in the CSFP group increased from the 2^nd^ week to the 12^th^ week in both groups; the mRNA expression levels of *OCG-3* in the CSFP group were significantly higher than those in the Non-CSFP group in the 8^th^ and 12^th^ weeks (*p* < 0.05) (Figure 10(k)).

Moreover, in the CSFP group, the protein expression levels of BMP-2 on the dural surface were higher than those on the paraspinal muscle surface in the 8^th^ week (Figure 10(b)). In the 12^th^ week, the undeveloped artificial laminae on the paraspinal muscle surface showed higher expression levels of BMP-2 than the developed artificial laminae on the dural surface (Figures 10(c), 10(g), and 10(h)).

## 4. Discussion

MSCs are among the most promising stem cell types for vascular tissue engineering, which can provide both seed cells and favorable cytokines for blood vessel formation [6]. Firstly, MSCs can be differentiated into several vascular cell phenotypes, including endothelial cells and smooth muscle cells. Secondly, MSCs can also secrete various angiogenic cytokines, such as VEGF, MCP-1, IL-6, and exosomes, which can promote the proliferation and migration of endothelial cells. The *in vivo* microenvironment of MSCs not only contains biochemical factors but also exerts biomechanical forces, which could influence their angiogenic ability [7]. In this study, we investigated the role of CSFP, a specific pulsation force, in the angiogenic ability of tissue-engineered laminae made by osteogenic MSCs and hydroxyapatite-collagen I scaffold.

The effect of shear stress or cyclic strain on different types of MSCs has been studied previously. For example, bone-marrow-derived MSCs from many species can differentiate into endothelial-like cells when they are stimulated through physiological shear stress or cyclic strain conditions [16–18]. Shear stress can induce MSCs to release VEGF-A, HGF, bFGF, and IGF-1 [19]. Besides, shear stress can also induce MSCs to release exosomes, which may serve as an essential mediator of angiogenesis by transferring genetic materials and angiogenic molecules [7]. In this study, we used the CSFP bioreactor to simulate rabbit CSFP and stimulate the osteogenic MSCs, finding that the CSFP could promote the expression of angiogenic factors of osteogenic MSCs. The animal study also found that the CSFP group expressed more angiogenic factors than the Non-CSFP group; moreover, in the CSFP group, the dural surface (closer to the mechanical stimulation of CSFP) of the newborn laminae also expressed more angiogenic factors than the paraspinal muscle surface at the 8^th^ week. These results showed that CSFP stress could promote the angiogenic activities of MSCs and the angiogenesis of the newborn laminae.

A previous study has shown that CSFP could promote the osteogenesis of newborn laminae [4]. In this study, we also determined several osteogenic markers, and the CSFP group also showed higher BMP-2, Osterix, and OCG-3 expressions than those of the Non-CSFP group. The lamina symphysis of the dural surface, which was closer to the mechanical stimulation of CSFP, preceded that of the paraspinal muscle surface. Thus, we made clear once again that CSFP stress could promote the osteogenesis, but whether the effect is direct or as a result of better vascularization is still unknown. The formation of blood vessels and bone is not isolated processes, and they are tightly coupled by a special vessel subtype (type H vessel), which highly expresses CD31 (PECAM-1) and Emcn [20]. Type H endothelial cells could regulate angiogenesis and osteogenesis through the Notch signaling pathway [21]. Further research efforts are thus required to explore the relationship between angiogenesis and osteogenesis. In this study, we also found that the protein expression levels of BMP-2 decreased in the 12^th^ week, while the mRNA expression levels of Osterix and OCG-3 still increased. In the 12^th^ week, the artificial laminae on dural surface realized symphysis, and there was a little expression of BMP-2 in this area; the BMP-2 mostly expressed on the paraspinal muscle surface, which still underwent bone formation; thus, the BMP-2 expression levels decreased compared to the 8^th^ week. The discrepancy between protein expression and mRNA expression is also commonly seen [22]. Moreover, from the 12^th^ week, the artificial laminae began the remodeling process, and bone resorption outweighs bone formation [3]. These might explain for the decrease of BMP-2 expression levels in the CSFP group.

There is a limitation in this study due to the different biomaterials used for *in vivo* and *in vitro* studies. In the previous studies [3, 4], we have successfully used microporous hydroxyapatite-collagen I scaffold to reconstruct the artificial laminae, so we continued to use it for the *in vivo* study. However, the bioreactor study requires the use of membrane biomaterials with elastic modulus, which could respond to the simulated CSFP stress. Besides, MSCs had to attach to the surface of the membrane in monolayer to ensure the consistency of CSFP stress on the cells. Microporous hydroxyapatite-collagen I scaffolds did not meet these requirements, so we chose the PLGA membrane with elastic modulus for the *in vitro* study.

## 5. Conclusion

In conclusion, CSFP stress could promote the angiogenic ability of osteogenic MSCs and thus promote the angiogenesis and osteogenesis of tissue-engineered laminae. The pretreatment of osteogenic MSC with the CSFP bioreactor may have important implications for vertebral lamina reconstruction with a tissue engineering technique.

## Figures and Tables

**Table 1:** Sequences of oligonucleotide primers used for quantitative real-time qRT-PCR.

Genes	Forward primer (5′-3′)	Reverse primer (5′-3′)
OSTERIX	GCA CGA AGA AGC CAT ACT C	TGA CAG AAG CCC ATT GGT
OCG3	CGG CTA CAC CAT TGG GAT GT	GCG GGA TCG ACA ATA GGG TT
VEGFA	TAA ACC CCA CGA AGT GGT GA	TGA CGT TGA ACT CCT CGG TG
PECAM1	AGA AGT GGA AGT GTC CTC GGT G	GAG CCT TCC GTC CTA GAG TAT CTG
ACTB	CCC GAC AGC CAG GTC ATC	GTT GAA GGT GGT CTC GTG G

## Data Availability

The datasets used during the current study are available from the corresponding author on reasonable request.
